# Case of the Successful Application of a Ligature to the Subclavian Artery, in a Case of Aneurism

**Published:** 1826-11

**Authors:** M. le Baron Dupuytren

**Affiliations:** the Hôtel Dieu, Paris.


### ANEURISM.

*Case of the successful Application of a Ligature to the Subclavian Artery, in a Case of Aneurism.* Treated by M. le Baron Dupuytren, at the Hôtel Dieu, Paris.*

Charles Lechevalier, thirty-seven years of age, was admitted into the hospital in February. He had a false consecutive aneurism of the left axillary artery. He had been taken prisoner in Spain, and, in endeavouring to make his escape, he received a wound on the posterior part of the left shoulder. A large quantity of blood was lost, and the man became senseless. The hemorrhage then ceased, and a simple dressing was applied to the wound. At the end of three weeks the wound was healed, and no blood was lost during the cure. Two months after the infliction of the wound, the patient perceived in the axilla a small pulsating tumor, the size of a hazel-nut. The colour of the skin was natural. At the expiration of two years, which were passed in hard captivity, the tumor had acquired the size of a hen's egg, and the pulsations were very powerful. The size of it rapidly increased, in consequence of the fatigue he was exposed to in returning by long marches to France, and it was soon as large as the head of a child at birth. The arm was kept at a distance from the body by

* Répertoire d'Anatomie et de Physiologie, No. ii. p. 381.

the swelling. He now suffered considerable pain, and was unable to work: he therefore repaired to Paris in order to obtain the best surgical advice.

The tumor was situated in the hollow of the axilla, and was now as large as the head of an infant a year old. It was irregularly round, unequal upon the surface, particularly upon the inferior and anterior part, and it was covered with blue and dilated veins. It was hard and shining, and at every point pulsated strongly, and synchronously with the heart. On the anterior and lower part, it was covered by the skin; on the anterior and upper part, by the great pectoral muscle. It ascended to the clavicle, and left no sensible interval between it and that bone. The scapula, the clavicle, and indeed the whole of the shoulder, were raised by the tumor. The arm was rather thinner and weaker than the limb on the opposite side. The heat and sensibility were natural. No pulsation could be detected in the radial or brachial arteries: while, on the contrary, the pulsations of the subclavian artery were very powerful, and, when compressed, the pulsation of the tumor was suspended. But, to cause this suspension, it was required to apply the finger strongly against the middle part of the clavicle, and to support it at the same time upon the first rib. It was evident that a rupture of the tumor would shortly take place, and that the patient must necessarily be lost.

After having maturely reflected upon the various plans which have been suggested by different authors in similar cases, M. Dupuytren determined upon the application of a ligature, which he conceived to be the only mode of treatment from which success could fairly be anticipated. In this instance, pressure was rendered impossible, from the bulk of the tumor. It was still to be determined in what manner, and at what part, the ligature was to be applied. It appeared that the only way of protecting the patient from hemorrhage during the operation, and from inflammation and suppuration, &c. of the sac afterwards, was to tie the artery above the aneurismal tumor. It was not possible to tie the axillary artery, in consequence of the size of the aneurism. It was necessary, therefore, to secure the subclavian artery, which, in its course on the left side, presents three distinct parts: 1st, from its origin from the aorta to its entering between the scaleni muscles; 2d, from the entrance of the vessel into the scaleni muscles until its exit from their substance; 3d, from this last point to the superior surface of the first rib: an important distinction, which has scarcely been pointed out by authors. The second point in the course of the artery appeared the most advantageous, as the vessel is admitted alone into the interval of the scaleni muscles, and is entirely separated from the subclavian vein, and from the plexus of the nerves of the arm. In taking, then, the anterior scalenus muscle for a guide, the artery may be tied without the risk of including any nerve in the ligature. M. Dupuytren, it appears, had contemplated the performance of this operation in an analogous case, ten years before. "Circumstances, however, which were then beyond his control prevented him from realising his project, and left to foreigners the glory of having first attempted, and of successfully applying this ligature."*

The operation being determined upon, and anxiously wished for by the patient, venesection was had recourse to, to empty the vessels, and to prevent the plethora and determinations of blood, to which the ligature so frequently gives rise when it is applied to the larger arteries.

The patient lying upon a bed, M. Dupuytren made an incision rather obliquely from above downwards, and from within outwards, about one inch above the clavicle. This first incision divided the skin, platysma myoides, the subcutaneous cellular tissue, and also three small vessels, which were immediately secured. The application of the ligatures caused considerable pain at the bottom of the throat. In continuing the operation, the surgeon came to the cellular tissue, and the glands which surround the artery, and the nerves of the brachial plexus. The external border of the anterior scalenus muscle was then sought for, and the muscle was completely divided near its insertion, by means of a blunt-pointed bistoury. The artery could now be perceived. The pulsation of it was with facility suspended, by the introduction of a finger to the bottom of the wound. A grooved silver probe, bent to about a quarter of a circle, was passed under the artery. A stilet, armed with a silk ligature, was passed along the groove of the probe, and drawn out on the opposite side. The ligature was then placed round the artery. To prove that the artery was fairly included, the two ends of the ligature were drawn a little upwards, and the fingers at the same time placed at the bottom of the loop which it formed. The pulsation of the artery ceased. No pain was given by the frequent repetition of this experiment, and the same result always succeeded. The ligature which was applied to the small arteries that were divided at the beginning of the operation gave considerable pain, but that upon the principal artery was not even felt by the patient. It cannot be doubted that this remarkable circumstance arose from the section of the anterior scalenus, which rendered it so easy to exclude every nerve from the ligature. The pulsation of the tumor immediately ceased. Convinced of the inutility, and even of the danger, of the "ligatures d'attente," M. Dupuytren did not make use of them. The patient did not lose two spoonsful of blood during the operation. The wound was dressed with mild ointment; the tumor was covered with resolvents; and the limb, placed upon a pillow, was enveloped in bags filled with warm sand.

* We believe Mr. Ramsden, of St. Bartholomew's Hospital, was the first surgeon who ever tied the subclavian artery by cutting above the clavicle. The operation was performed in November 1809. The patient died five days afterwards. For a detail of this case, and some interesting observations upon the probable causes of the failure of the operation, the reader may refer to Cooper's Surgical Dictionary, p. 103, art. Aneurism. (Translator.)

In the course of the day, the patient complained of slight pain in the throat. A precautionary bleeding was had recourse to. There was no pulsation in the tumor, and he passed a good night. The limb was of the natural temperature, and retained its muscular powers and sensibility. A slight darting sensation was felt in the tumor.

On the eleventh day, the ligature came away, without the slightest hemorrhage. The size of the tumor began gradually to diminish a few days after the operation. On the thirtieth day, the wound was nearly healed, and the patient began to make some use of his arm. The tumor was soft and fluctuating, from which it was apprehended that suppuration and a spontaneous rupture might occur. It was kept constantly covered with compresses wetted with Goulard water, which were renewed every two hours. By the seventy-eighth day, the tumor was of a much firmer consistence, and suppuration was no longer feared. The heat, sensibility, and muscular power were equal to those of the right arm. The circulation of the limb had that peculiar character which is always observed when the principal artery has been tied. No pulsation could be detected in the arteries. It was evident to the touch that they were full, and that the blood proceeded through them; but, in passing through the numerous and minute anastomoses which conducted it from the superior to the inferior parts of the limb, the blood had ceased to be influenced by the action of the heart, which produces in the other parts of the arterial system that alternate dilatation and contraction which constitute the pulse.*

A few months afterwards, Chevalier resumed his business of a joiner. During three years he continued free from complaint, and no longer thought of the disease from which he had so fortunately escaped. In consequence, however, of great exertion, inflammation took place in the axilla. Being uncertain of the nature of the complaint, he returned to Paris. A tumor, about the size of the fist, was found in the axilla; it was pointed, and threatened to break. No pulsation was to be detected in it. Emollient applications were used, as it was clear the tumor had no connexion with the circulation of the blood, and at the end of fifteen days it spontaneously ruptured. A large quantity of pus was discharged, coloured with blood, which had long remained in the swelling: not a drop of red or arterial blood was discharged. The opening was enlarged by M. Dupuytren, with a probe-pointed bistoury. The suppuration gradually diminished; the sides of the tumor approached each other, and the patient was sent from the hospital perfectly cured. The axilla was perfectly free from enlargement of any sort.

The distinguishing points of this operation from others in which the subclavian artery has been tied, are the spot in

* This is a question still *sub judice*. Vide "An Experimental Inquiry into the Nature and Causes of the Arterial Pulse," by Dr. Parry. (Translator.)

which the ligature was applied, and the section of the anterior scalenus muscle. By this means the surgeon is sure to discover the artery, in following the external margin of that muscle, and of avoiding the adjacent veins and nerves. It is presumed by the relator of the case, M. Marx, that, in future, the above method of performing this important operation will certainly be adopted. Two plates are given, which represent the situation and volume of the tumor before the operation, and the ligature around the subclavian artery, between the two scaleni muscles.

